# Normal Ovariotomy

**Published:** 1873-06

**Authors:** J. T. Gilmore

**Affiliations:** Mobile, Ala.


### EDITORIAL AND MISCELLANEOUS.

---

### NORMAL OVARIOTOMY.

The new operation of normal ovariotomy is engaging the thought and comment of the profession so generally at the present time as to make every thing bearing upon the subject of interest to our readers.

It affords us pleasure to be able to put upon record some additional facts, derived from a second case operated upon by Prof. J. T. Gilmore, of Mobile, Alabama, and kindly furnished us in the subjoined letter from that skilled surgeon:

MOBILE, ALA., June 15, 1873.

*Dear Doctor*—Your letter relative to a case of normal ovariotomy was received some days ago.

The case I operated upon was unlike yours, in having an atrophied uterus of many years duration. Whilst the menstrual irritation had, since the menopause, been productive of great discomfort, it was only within the six months immediately preceding the operation that it had threatened life by the violent congestions of the brain which were recurring regularly every two weeks at the period of the operation, at which time she had become almost idiotic.

She was in bad plight for any operative procedure at the time it was done, and yet, so threatening were the paroxysms, it was feared that another one would destroy her life.

Drs. Ketchum and Gaines had attended her for years and had resorted to every resource in the effort to reëstablish the menstrual flow, but without avail, and they both assented to the proposition that if normal ovariotomy was a legitimate operation under any circumstances, the case in question justly claimed its benefits. Dr. Ketchum assented to the operation. Dr. Gaines hesitated, but his objection presented no real basis upon which to ground an opposition. Drs. W. H. Anderson,

Ketchum, and Gaines discussed the probable physiological effect of a removal of the ovaries, and all agreed that without some snch intervention the patient would certainly not get through with many more such attacks as she had recently endured.

Dr. Anderson, who fills the Chair of Physiology in our Medical College, urged the operation upon physiological grounds. The absence of the uterus rendered it improbable that there could ever be any more menstrual elimination, and the purpose of the operation would be to destroy, as far as possible, the menstrual irritation, by putting a stop to ovulation.

The operation was done in December last.

The peritonitis that was lighted up, and the celiulitis which followed, ended in abcess, which is still discharging through the abdominal wall, at the point of incision. The patient barely escaped with her life.

Her brain is now relieved. Every two weeks she has still some hysterical symptoms, *i. e.*, choking sensations, with the feeling as though there were some foreign body in the throat. There has been no menstrual show whatever. There is still discharging from the abdominal fistula about an ounce and a half of thin pus daily, and the cavity of the abcess I wash out every third or fourth day with carbolized water, through a catheter and syringe. The abdominal discharge is lessening daily, and there are noticeable changes in her features and mammary glands.

I shall await the full results of the case, before publishing it in detail.

This is an important subject you have opened up to the profession, and deserves a most honest and careful detail of cases operated.

Yours truly, J. T. Gilmore.

We hope, at a proper time in the future, to be able to lay before our readers the full particulars of this case, the operation and its results, both physiological and curative. B.

